# Next-generation sequencing and PCR technologies in monitoring the hospital microbiome and its drug resistance

**DOI:** 10.3389/fmicb.2022.969863

**Published:** 2022-07-28

**Authors:** Carolina Cason, Maria D’Accolti, Irene Soffritti, Sante Mazzacane, Manola Comar, Elisabetta Caselli

**Affiliations:** ^1^Department of Advanced Translational Microbiology, Institute for Maternal and Child Health, IRCCS “Burlo Garofolo”, Trieste, Italy; ^2^Department of Chemical, Pharmaceutical and Agricultural Sciences, Section of Microbiology and LTTA, University of Ferrara, Ferrara, Italy; ^3^CIAS Research Centre, University of Ferrara, Ferrara, Italy; ^4^Department of Medical Sciences, University of Trieste, Trieste, Italy

**Keywords:** NGS, microbiome, resistome, healthcare-associated infections, hospital environment

## Abstract

The hospital environment significantly contributes to the onset of healthcare-associated infections (HAIs), which represent one of the most frequent complications occurring in healthcare facilities worldwide. Moreover, the increased antimicrobial resistance (AMR) characterizing HAI-associated microbes is one of the human health’s main concerns, requiring the characterization of the contaminating microbial population in the hospital environment. The monitoring of surface microbiota in hospitals is generally addressed by microbial cultural isolation. However, this has some important limitations mainly relating to the inability to define the whole drug-resistance profile of the contaminating microbiota and to the long time period required to obtain the results. Hence, there is an urgent need to implement environmental surveillance systems using more effective methods. Molecular approaches, including next-generation sequencing and PCR assays, may be useful and effective tools to monitor microbial contamination, especially the growing AMR of HAI-associated pathogens. Herein, we summarize the results of our recent studies using culture-based and molecular analyses in 12 hospitals for adults and children over a 5-year period, highlighting the advantages and disadvantages of the techniques used.

## Introduction

It is generally recognized that built environments (BE) can be considered super-organisms, with their own microbiome that, in more confined environments, show more anthropic origin, less biodiversity, and higher antimicrobial resistance (AMR; Mahnert et al., 2019).

In the hospital environment, these features are very important because the hospital microbiome represents the reservoir for the so-called healthcare-associated infections (HAIs), which are a concern worldwide (Green et al., 1998; Dancer, 2008; Mahnert et al., 2019) and are the most frequent and severe complications of hospitalization (Dancer, 2014). Healthcare-associated infections affect more than four million people per year in the European Community alone, causing over 37,000 deaths (ECDC, 2013, 2015; EARS, 2017).

The hospital microbiome, derived from patients, healthcare workers, and visitors, persists in the hospital environment and thus becomes a reservoir of pathogens and a source of infection (Riggs et al., 2007). Accordingly, the risk of acquiring an infection is increased if a patient is hospitalized in a room previously occupied by an infected patient (Huang et al., 2006; Drees et al., 2008; Datta et al., 2011). This has been reported for *Staphylococcus aureus* (Boyce et al., 1997), *Enterococcus* spp. (Huang et al., 2006), *C. difficile* (Samore et al., 1996), and *Acinetobacter* spp. (Getchell-White et al., 1989). Microorganisms can persist for extended periods on inanimate surfaces, increasing the risk of colonization for hospitalized patients (Kramer et al., 2006). In particular, the ESKAPE pathogens, responsible for severe HAIs (*Enterococcus faecium, S. aureus, Klebsiella pneumoniae, Acinetobacter baumannii, Pseudomonas aeruginosa,* and *Enterobacter* species) and *Clostridium difficile* are all able to survive for particularly extended periods of time in the healthcare environment (Pendleton et al., 2013).

Healthcare-associated infection severity is tightly associated with AMR concern, which has been continuously increasing in previous decades. Increased antimicrobial resistance of pathogens is particularly frequent in the hospital environment, where the fast rise of microbial resistance is both favored by the selective pressure exerted by the continuous and increased use of antimicrobial drugs and disinfectants (Kampf, 2018); and is also driven by the transfer of AMR genes between taxa through lateral gene transfer (LGT), which represents one of the most dramatic and detrimental consequences of anthropogenic impacts on the evolution of other species (Lax and Gilbert, 2015).

Furthermore, HAIs **c**an be caused by fungi as well as bacteria (Boyce et al., 1997; Weber et al., 2010, 2013; Dancer, 2014), which also start to exhibit drug-resistance, demonstrating that all these microbial components should be controlled to counteract AMR and HAI concerns (D’Accolti et al., 2022). Surveillance appears to be a key component of control strategies, because a deep knowledge of the hospital microbiome may enable real-time countermeasures to prevent AMR spread and possible HAI outbreaks, as well as enable monitoring of the effectiveness of sanitation strategies.

## Monitoring the environmental microbial contamination: Culture-based and molecular approaches

Microbiological contamination can be assessed by different methods, one of which is adenosine triphosphate (ATP) bioluminescence (D’Accolti et al., 2019). Although it is used often due to its low cost and rapidity, it has low sensitivity and is unspecific, detecting not only microbes but also organic materials (Dancer, 2014). Thus, this method may be useful for determining the cleanliness of surfaces but it is not effective for assessing the degree of sanitation and the microbiological composition of the hospital microbiome, which is important to guide AMR and HAI prevention (Mitchell et al., 2013; Nante et al., 2017). Other widely used methods are based on culture isolation and CFU (colony forming unit) count. These methods have been used for decades in healthcare settings, though mostly for high-risk area monitoring (Dancer, 2014; D’Accolti et al., 2019). Sampling is usually performed by RODAC plates (Replicate Organism Detection and Counting plates, containing general or specific selective media), dip slides, nitrocellulose membranes, swabs, sponges or wipes able to collect microbes from surfaces. The samples are incubated for the appropriate time (2–7 days) at the appropriate temperature that allows the development of colonies, which are finally counted and provide a quantitative measurement of the contamination degree of specific species. Although effective, these methods are very time-consuming and are only effective for living microorganisms whose growth conditions can be reproduced in the laboratory. Thus, culture-based methods do not provide detection of unsampled or non-cultivable microbes, consequently underestimating the level of microbiological contamination and complexity (Christoff et al., 2019). Moreover, further tests are needed for species identification (i.e., biochemical or immunological tests) and for the assessment of drug susceptibility/resistance profile of the isolates, which is usually performed by manual or automated diffusion or dilution tests.

Molecular methods, being based on the analysis of the genetic material of the sample, are characterized by high rapidity, sensitivity, and specificity, allowing the detection and quantification of even a very low number of targets in a few hours, and providing an in-depth characterization of the microbial community (D’Accolti et al., 2019). These methods include qualitative and quantitative real-time polymerase chain reaction (PCR, qPCR) and qPCR microarray and can provide the microbial profile of the sample and its AMR characterization due to the presence of AMR genes that can predict the phenotypic AMR in the contaminating microbiome (Hendriksen et al., 2019).

**Figure 1:**
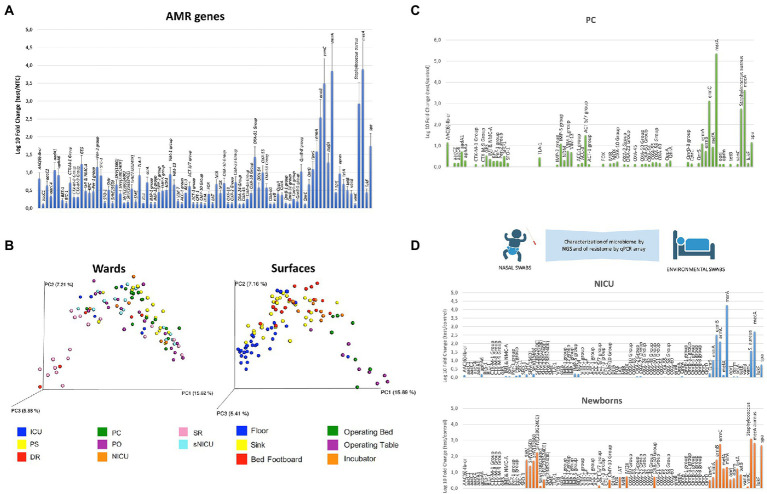
Characterization of the hospital microbiome and its AMR. **(A)** Detection of AMR genes and *Staphylococcus aureus* presence by real-time quantitative (qPCR) microarray. The results represent the mean values detected in the General Medicine wards of 12 Italian hospitals from 2015 to 2020, and are expressed as mean ± SE. Log_10_ fold change values for each gene compared to negative controls. In addition to AMR genes, *S. aureus* and two of its virulence factors (*spa* and *lukF*) are also identified by the microarray. **(B)** NGS characterization of the microbial communities residing on different types of surfaces in the wards of a pediatric hospital. The unifrac-based principal coordinates analysis (PCoA) plots show the clustering of bacterial communities according to the wards **(A)** and surfaces **(B)** investigated. Each dot represents a sample. PC, Pediatric Clinic; PS, Pediatric Surgery; NICU, Neonatal Intensive Care Unit; sNICU, sub-Intensive Care Unit; ICU, children’s Intensive Care Unit; SR, Surgical Rooms; DR, Delivery Room; PO, Pediatric Oncology. **(C)** Detection of AMR genes by qPCR microarray in the Pediatric Clinic ward of a children’s hospital. The results are expressed as log_10_ fold change values compared to negative controls. **(D)** Colonization of preterm infants by Neonatal Intensive Care Unit (NICU) environmental microorganisms; the AMR analysis was performed by qPCR microarray. The results are expressed as log_10_ fold change values compared to negative controls, relative to the NICU environment (NICU) and to the newborn’s nasal cavity after 2 weeks of hospitalization in NICU (newborns).

In recent years, many specific qualitative PCR, qPCR, and qPCR microarrays have been developed and commercialized, which have been used to describe surface bioburden in a very rapid and specific manner, compared to culture-based approaches (Caselli et al., 2016, 2018, 2019a). We have previously reported the use of qPCR microarrays to assess the AMR profile of the microbiome of several Italian hospitals, providing information on AMR and its fluctuations over time or as a result of the application of specific sanitation procedures (Caselli et al., 2016, 2019a, 2020; Comar et al., 2019; Cason et al., 2021). Figure 1A summarizes the microbiome detected in the general medicine wards of 12 Italian hospitals enrolled in studies from 2015 to 2020, including public and private settings with over 100 beds, located in different geographical regions and sampled during different periods of the year. The results, independent of the location and sampling time, showed the presence of persistent contamination by ESKAPE pathogens together with the presence of several AMR genes, conferring resistance to almost all classes of available antibiotics. In particular, *Staphylococcus* spp. was identified as a prevalent contaminant, followed by Gram-positive *Enterococcus* spp., Gram-negative *Enterobacteriaceae,* and fungi (*Candida* and *Aspergillus* spp.; Caselli et al., 2016, 2018). There was a high prevalence detected of the gene conferring the resistance to methicillin (*mecA*) in all sampled settings. The high prevalence of *mecA* was likely due to the high prevalence of *S. aureus* that was also detected on the sampled surfaces (Figure 1A). This was identified by the specific qPCR which was included in the microarray due to previous results, showing that *Staphylococcus* genus was the most prevalent among hospital surface contaminants (Caselli et al., 2016, 2018). The microarray also included two virulence genes characterizing *S. aureus* among the other *Staphylococcus* species with coding for Staphylococcal protein A (*spa*), which is an important virulence factor enabling *S. aureus* to evade host immune responses (Votintseva et al., 2014), and Leucocidin F (*lukF*), a toxin leading to trans-membrane pore formation (Pedelacq et al., 1999). Both of these genes have been consistently detected together with *S. aureus* and the *mecA* gene. Other prevalent AMR genes detected on hospital surfaces included *msrA* (coding for a macrolide efflux protein present in a wide bacterial host range), *ermA-B-C* (coding for resistance to erythromycin, lincosamides, and streptogramin), *OXA-51 group* (conferring resistance against beta-lactams and carbapenems), and *QnrS/QnrB-8 group* (coding for resistance against quinolones). By using a sanitation procedure able to modulate the stability of the hospital microbiome compositions—based on the competitive exclusion exerted by *Bacillus* probiotics included in the cleaning system—a significant decrease (80%) in pathogens was observed in the treated environments, accompanied by a halving of the HAI incidence (Caselli et al., 2018). This highlighted the contribution of the hospital environment to HAI acquisition. The same environmental strains decreased due to the introduction of probiotic sanitation and also decreased as etiological agents isolated from HAI patients, confirming the relationship between the persistence of the resistant hospital microbiome and HAI occurrence (Caselli et al., 2018, 2019a).

This approach can be introduced to control, in a timely manner, any change/acquisition of new AMR genes by the hospital microbiome, thus inhibiting the spread of AMR and difficult to treat HAIs. However, PCR-based methods cannot describe the whole microbiome and/or resistome, being targeted to specific microorganisms and genes. An efficient monitoring system should provide a deep characterization of the environmental bioburden, providing rapid and detailed information on the microbial population, to enable prompt monitoring of any eventual changes in microbial communities and in their AMR.

Based on these considerations, metagenomics studies involving deep-sequencing approaches have been recently identified as powerful tools. They have the potential to provide an in-depth characterization of the microbial community, and to obtain an exhaustive picture of the environmental microbiome and its interaction with the human beings inhabiting that environment.

## The deep-sequencing approach: NGS and WGS

Recent advancements in DNA sequencing technologies have dramatically improved microbiological diagnosis as well as microbiological surveillance. In early metagenomic studies, the use of Sanger sequencing technology provided important advances in the knowledge of microorganisms and of their functions (Sanger et al., 1977; Gillespie et al., 2002; Breitbart et al., 2003). However, the advent of next-generation sequencing (NGS) technologies has enormously strengthened the field, enabling simultaneous sequencing of millions of DNA fragments at a lower cost than Sanger sequencing (Kircher and Kelso, 2010). Consequently, in the last decades, NGS has not only provided a deeper knowledge of the human microbiome but has greatly increased our understanding of the microbiome of different habitats including built environments. It has enabled the detection of non-cultivable microbes and has revealed new insights into indoor microbial spread. Moreover, being not limited to targeted microorganisms, NGS has provided a thorough picture of the sampled microbial population, revolutionizing our ability to study hospital-associated bacteria due to the availability of several accessible bioinformatics resources, both for microbiome and AMR studies (Hendriksen et al., 2019). Next-generation sequencing has been recently introduced for the characterization of the microbiome of hospital surfaces, identification of novel pathogens, tracking of disease outbreaks, and the study of AMR evolution (Zhong et al., 2021). Despite the existence of different NGS platforms and sequencing technologies, all NGS procedures are able to simultaneously sequence high numbers of DNA fragments, which are subsequently mapped by bioinformatic analyses through comparison with reference genomes (Behjati and Tarpey, 2013). Next-generation sequencing can be used to sequence entire genomes or target specific genes. This enables it to reveal and trace specific pathogens and overcome the limitations of conventional characterization of pathogens by its morphological, staining, and metabolic features. The genome of microbes also contains information on drug susceptibility/resistance; therefore, the NGS technique can be used to detect possible sources of difficult-to-treat infections, allowing timely interventions and possible benefits for patient care. This approach shows strong potential for accurate and rapid identification of the transmission pathways between hospital and community settings, and has been used to monitor MRSA outbreaks, for example (Harris et al., 2013).

Deep-sequencing applications can be applied to hospital surveillance and can include amplicon sequencing, whole metagenome shotgun analyses, and RNA sequencing. RNA sequencing is a valid and promising method for microbial characterization, and can be used to determine the transcriptome of a microbial population. This is a further step toward defining microbiome functions, the two main NGS methodologies currently in use are amplicon sequencing and shotgun metagenomic sequencing (Wensel et al., 2022).

Amplicon metagenomic sequencing is an ultra-deep DNA sequencing method that focuses on specific target regions (amplicons). Short hypervariable regions of <500 base pairs (bp) harbored in conserved genes are amplified by PCR using universal primers targeting bacterial 16S ribosomal RNA (rRNA), fungal 18S rRNA, or Internal Transcribed Spacer (ITS) rRNA. The subsequent sequencing of the amplified fragments can provide the characterization of complex microbial communities, as well as the identification of individual species and detection of microorganisms of interest among many others. Furthermore, besides the identification and tracking of microorganisms of interest, 16S/18S/ITS rRNA sequencing data can be used to describe and compare the diversity of multiple complex environments through Alpha (α) and Beta (β) diversity analyses, providing useful parameters for comparing complex microbial communities.

This sequence-based approach, when applied to the study of AMR, can provide information on all AMR genes and their precursors in a microbial species or population, thus profiling the whole resistome (Zankari et al., 2013). In recent years, several studies have been published describing the use of NGS-based methods for AMR profiling in clinical, food, and environmental contexts (Sherry et al., 2013; Bengtsson-Palme et al., 2014; Bradley et al., 2015; Hasman et al., 2015; Noyes et al., 2016; Votintseva et al., 2017), and were acknowledged by the European Commission coordinating the action plan against AMR (Angers et al., 2017). Next-generation sequencing panels for targeted sequencing are now available commercially, mainly based on amplicon sequencing techniques (including Ion AmpliSeq by Thermo Fisher, AmpliSeq by Illumina, and others).

In the shotgun metagenomic approaches, the extracted DNA is first fragmented by shotgun, generating shotgun libraries that are sequenced, thus providing unrestricted sequencing of all the microbial genomes present in a sample. Shotgun metagenome sequencing is currently performed for taxonomic profiling (diversity and abundance), allowing for parallel sequencing of DNA from all organisms within a community, with high coverage for species-level detection. The resultant sequencing reads are then aligned to a reference database to identify taxa. Thus, being independent from amplification, shotgun metagenomics allow the identification—in a unique sample and with improved genus and species level classification—of bacteria, archaea, eukaryotes, and DNA viruses, with broad taxonomic coverage, accurate functional profiling, and the possibility of detecting previously unknown species and strains of microbes.

Shotgun metagenomics have also been proposed for the detection of AMR-related genes (Bengtsson-Palme et al., 2017; Crofts et al., 2017), since this technique is able to simultaneously quantify thousands of transmissible AMR genes in a single sample, without any prior selection of specific genes to look for. Furthermore, it can provide information about the presence of specific bacterial species and virulence genes, and later, the data can be re-analyzed if needed. In recent years, the reduction in cost and increase in the quality of whole-genome sequencing (WGS) has rendered this technique affordable and feasible as a routine tool, not only in diagnostics but also for surveillance (Aarestrup et al., 2012). Genotyping using WGS to define antimicrobial susceptibility has been extensively reported for bacterial isolates, showing high concordance with phenotypic susceptibility assays (Stoesser et al., 2013; Zankari et al., 2013; Shelburne et al., 2017). However, in 2017, the EUCAST Committee reported a lack of sufficient evidence to support the use of WGS to guide clinical decisions, also identifying the high-cost and limited rapidity of inferring AMR from WGS data (Ellington et al., 2017). Moreover, an obstacle to its routine use is the generation of high amounts of bioinformatics data to interpret, rendering the provision of real-time data time-consuming for clinical decisions (Jovel et al., 2016; Wensel et al., 2022).

Taking into consideration the advantages and disadvantages of the different metagenomics approaches, most of the studies aimed at characterizing the hospital microbiome and its AMR for HAI prevention are currently based on amplicon sequencing (Poza et al., 2012; Bokulich et al., 2013; Hewitt et al., 2013; Oberauner et al., 2013; Tang et al., 2015; Rampelotto et al., 2019; Pochtovyi et al., 2021; Perry-Dow et al., 2022), whose main objective is to identify the microorganisms circulating in a certain environment at a certain time, limiting costs, and bioinformatics concerns (Jovel et al., 2016). In particular, the 16S rRNA sequencing has been mostly used for hospital monitoring, due to the high prevalence of bacteria in the hospital microbiome. The 16S rRNA gene is in fact conserved in bacteria but also contains hyper-variable regions (V1–V9) that allow differentiation between bacterial genera and species, by comparison against reference databases such as GenBank and RDP (Christoff et al., 2019). Metagenomics has been broadly applied for the study of the resistome of the human and animal microbiome (Afridi et al., 2020, 2021; Caselli et al., 2020), and of that from different habitats, including agricultural and urban soils (Crofts et al., 2017). It has also been applied for local and international AMR surveillance of specific strains, mostly associated with HAI onset, such as carbapenem and quinolone-resistance Gram-negative bacteria, multi-resistant *S. aureus* and *Enterobacterales*, and *M. tuberculosis* (Soliman et al., 2020; Lebeaux et al., 2021; Vazquez-Lopez et al., 2021; Waddington et al., 2022). Interestingly, by using a metagenomic approach, it was possible to show that more confined and cleaned environments had reduced microbial diversity and increased resistance, suggesting the implementation of strategies to restore bacterial diversity in certain built environments (Mahnert et al., 2019). Less data are available on the whole resistome of the microbial population colonizing the healthcare facilities, despite the high prevalence of drug-resistant isolates in this kind of environment. This is due to omnipresent selective pressure that is correlated with the extensive use of antibiotics and antimicrobials, which may co-select for antibiotic resistance (Crofts et al., 2017; Kampf, 2018). Interestingly, an AMR analysis of hospital and community wastewater, conducted in 23 countries, demonstrated a higher amount of AMR genes in hospital wastewater, suggesting an important role for hospital effluent as a source of AMR spread in the environment (Hassoun-Kheir et al., 2020).

In recent years, our group has applied the 16S rRNA NGS approach in various studies, to analyze the hospital microbiome in the different types of wards of a pediatric hospital. We compared NGS data with those obtained by conventional culture-based methods and PCR-based techniques (Comar et al., 2019; Cason et al., 2021; Soffritti et al., 2022). In the first study, over 100 surface critical points were analyzed by sequencing the V3 hypervariable region of the16S rRNA, and in parallel by culture-based techniques (RODAC contact plates) and by qPCR microarray, to characterize the advantages and disadvantages of every method. The results showed that the higher sensitivity of the molecular methods compared to culture-based ones (Comar et al., 2019), provided a more accurate quantification of the actual microbial contamination and enabled the identification of different microbial communities in the diverse types of wards and surfaces (Figure 1B). The16S rRNA NGS method was uniquely characterized by its ability to detect unsearched bacteria; however, it did not detect mycetes, which were identified and quantified by the culture-based and targeted PCR methods. The analysis of the AMR genes harbored by the complex microbial population colonizing the sampled surfaces, performed by a qPCR-microarray simultaneously detecting 81 AMR genes, allowed the sketching of the main AMR features of the microbial communities colonizing the different types of wards (Comar et al., 2019). A higher presence of AMR genes was detected in general medicine wards (such as the Pediatric Clinic; Figure 1C) compared to more controlled areas with less microbial contamination (Surgical and Delivery Rooms). This confirmed the results of studies performed in adult hospitals, where general medicine wards were identified as the most contaminated areas. This approach also identified significant qualitative and quantitative differences between the pediatric and the adult wards that were likely due to the different drugs used, which exerted a different selective pressure on the hospital microbiome. Based on these results, the same approach was used to analyze the evolution of the nasal microbiome of preterm newborns recovering in the neonatal intensive care unit (NICU; Figure 1D; Cason et al., 2021). The infants’ nasal microbiome was analyzed by NGS and qPCR microarray at delivery as well as during NICU stay. This was compared with the microbiological composition of the mother’s vaginal tract and from the surfaces of the delivery room and NICU environment. This analytical approach identified a prompt colonization of the newborns’ nasal cavities by the NICU environment microbes (including AMR strains). These were absent just after birth but increased with the duration of NICU stay (up to 2 weeks of hospitalization), suggesting that the hospital surface microbiota was not only responsible for contact-transmitted HAIs, but it may also have been transported by air particles to reach the respiratory tract (Cason et al., 2021).

Recently, the combined NGS/qPCR analysis was used to characterize the microbiome of the Emergency Room during the COVID-19 pandemic. The results showed that the type of sanitation adopted changed the microbiome of the hospital environment, in terms of both the amount of pathogen and the prevalence of AMR (Soffritti et al., 2022), thus prompting a re-think of the sanitation approach in order to limit AMR spread and HAI onset. Taken together, the collected data highlight the importance of active environmental monitoring to define AMR features of the hospital microbiome in depth in order to counteract more efficiently AMR spread and HAI concerns.

Compared to the results obtained by qPCR microarray, the use of NGS may deepen the resistome analysis, allowing more exhaustive profiling of the AMR features of the analyzed microbiome. The microarray-based results obtained by analyzing the whole contaminant population were in accord with the published NGS/WGS studies on hospital isolates, showing high amounts and prevalence of AMR genes coding for resistance against beta-lactams (including methicillin and carbapenems), quinolones, macrolides, erythromycin, lincosamides, and streptogramin (Waddington et al., 2022). This strengthens the notion that the hospital environment can be a reservoir of resistant HAI pathogens that can be transmitted to patients. Limitations to the use of NGS include the resolution of NGS being lower than WGS, thereby limiting the taxonomic resolution of closely related species (Jenkins et al., 2012), and that different studies usually report only a subset of targeted variable regions, thereby requiring standardized protocols to avoid differing data interpretation (Chakravorty et al., 2007). However, metagenomics represents a culture-unbiased tool to obtain information on the genes responsible for AMR and on the microorganism harboring such genes (Kristiansson et al., 2011; Snitkin et al., 2012; Bengtsson-Palme et al., 2016b). Assembling the sequences into longer stretches of DNA to reconstruct complete genes allows the detection of AMR genes and provides information on the clonal lineage of different isolates, leading to the discovery of new resistance patterns and completely novel resistance plasmids, as well as detecting changes in taxonomic composition and other functional genes. The ability of a gene to confer resistance and to spread to other bacteria varies according to the gene’s context, for example, if it is located in regions involved in horizontal gene transfer (Kristiansson et al., 2011; Bengtsson-Palme et al., 2016a).

**Figure 2:**
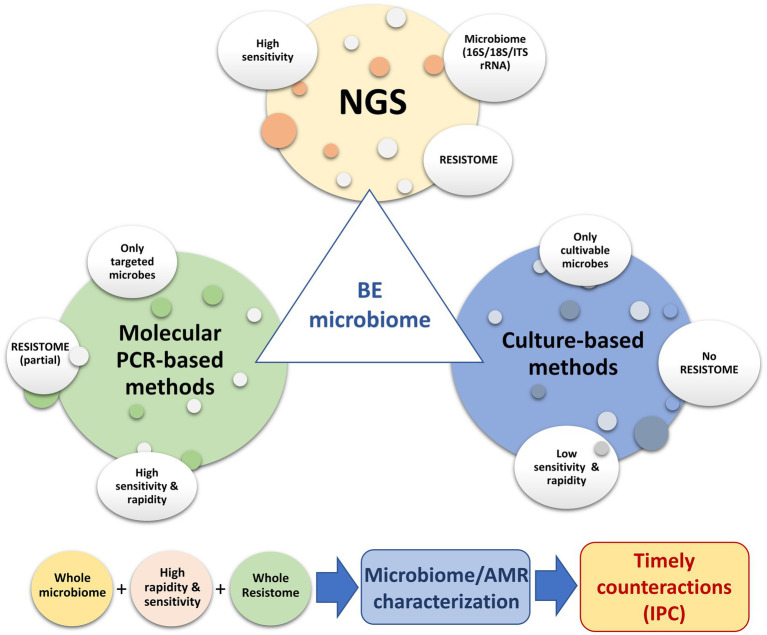
Use of different molecular and conventional methods to depict the microbiome of the hospital environment, with particular focus on AMR surveillance.

Metagenomics may result in low sensitivity due to sequencing all of the DNA present in the sample. By contrast, quantitative PCR methods may provide higher sensitivity. A direct comparison of these methods, with respect to sensitivity and specificity, is needed. Next-generation sequencing represents an invaluable tool for the in-depth characterization of the resistome of hospital surfaces. However, for daily surveillance practice, where it is necessary to have results in a short period of time and at reduced costs, the applications of molecular methods such as qPCR microarray—that identify and quantify selected panels of resistance genes associated with HAIs—have been shown to be effective and may be easier to apply (Caselli et al., 2016, 2018, 2019b; Cason et al., 2021). The higher detection limit of shotgun metagenomics compared to qPCR, particularly when only a few million reads are generated per sample, should also be considered (Bengtsson-Palme et al., 2017). The main limitation of molecular methods, such as qPCR microarray, is the lack of information provided on the bacterial hosts of the resistance genes; however, the detection of these genes appears to be valuable information and should not be underestimated, for example, the phenomena of LGT. A combined approach may provide a complete picture, allowing timely countermeasures to be taken, if necessary (Figure 2).

## Conclusion

During the last decade, metagenomics approaches have significantly improved analysis capacities, and the cost of new options available to scientists and clinicians is constantly decreasing. The high-throughput sequencing of 16S rRNA gene is uniquely able to identify unsearched bacteria and AMR genes, compared to targeted PCR and conventional microbiological methods, rendering it an effective first-step tool for monitoring the hospital microbiome and evaluating the safety of the environment, in terms of associated infectious risk. Next-generation sequencing can identify all the genetic material in a sample even if present in trace amounts. Due to the LGT of AMR among bacteria (Lax and Gilbert, 2015) and the low infectious dose necessary to trigger an infection (Dancer, 2014), the presence of traces of microorganisms or resistance genes should not be underestimated in this type of monitoring. The control of AMR can benefit from NGS at various levels; it has rapidly become a reference method for studies on genomic epidemiology, clinical metagenomics, and environmental analyses, where it can help in tracking multidrug-resistant microbes and their transfer to humans.

The presence of traces that do not necessarily belong to viable microbes at the time of sampling may present a bias. This could be overcome by treating the sample with PMA (propidium monoazide) to block the amplification of dead cells’ DNA, for example (Vaishampayan et al., 2013; Emerson et al., 2017).

Furthermore, 16S rRNA-based NGS cannot detect eukaryotic cells, such as fungi, or distinguish virulent from non-virulent bacterial species. Therefore, the addition of other molecular tools, such as WGS or specifically targeted PCR, should be considered in order to obtain sensitive and accurate identification of pathogens and their AMR.

Currently, qPCR microarrays seem to be a useful tool for monitoring the hospital environment, due to their rapid results on the AMR genes of interest, less complex data processing requirement compared to NGS or WGS, and less expensive instruments (thermal cyclers; Caselli et al., 2016; Comar et al., 2019; Cason et al., 2021; Soffritti et al., 2022). In the studies of several Italian hospitals, this monitoring approach was successfully used to characterize the hospital microbiome and its AMR before and after the introduction of a sanitation system. It was able to stably shape them, resulting in a significant decrease in the environmental resistant pathogens and in their isolation from HAI patients. This confirmed the important contribution of the hospital environment to HAI occurrence (Caselli et al., 2018, 2019a).

In conclusion, currently available data suggest that NGS bacteriome analysis together with a similar evaluation of the mycobiome (by 18S rRNA analysis) and of the resistome of the microbial population, may provide a deeper knowledge of the microbiome contaminating the hospital environment. This will lead to consistent improvements in protocols and interventions to counteract the increasing AMR diffusion. Molecular analyses can be conveniently included in surveillance programs, providing standardization of pipelines and interpretation of the genomic data, which is urgently needed. In the near future, using these molecular investigations, our understanding of the resistome may become sufficiently mature to enable the development of strategies that actively fight resistance mechanisms and stop the spread of AMR.

## Author contributions

CC, MD’A and EC performed literature search, drafted the manuscript, and prepared the figures. IS, MC, and EC edited and revised the manuscript. EC initiated, supervised, and finalized the work for submission. All authors contributed to the article and approved the submitted version.

## Conflict of interest

The authors declare that the research was conducted in the absence of any commercial or financial relationships that could be construed as a potential conflict of interest.

## Publisher’s note

All claims expressed in this article are solely those of the authors and do not necessarily represent those of their affiliated organizations, or those of the publisher, the editors and the reviewers. Any product that may be evaluated in this article, or claim that may be made by its manufacturer, is not guaranteed or endorsed by the publisher.
